# Author Correction: Membrane curvature governs the distribution of Piezo1 in live cells

**DOI:** 10.1038/s41467-023-36108-9

**Published:** 2023-01-31

**Authors:** Shilong Yang, Xinwen Miao, Steven Arnold, Boxuan Li, Alan T. Ly, Huan Wang, Matthew Wang, Xiangfu Guo, Medha M. Pathak, Wenting Zhao, Charles D. Cox, Zheng Shi

**Affiliations:** 1 Department of Chemistry and Chemical Biology, Rutgers University, Piscataway, NJ 08854 USA; 2 School of Chemistry, Chemical Engineering, and Biotechnology, Nanyang Technological University, 637457 Singapore, Singapore; 3 Department of Cell Biology and Neuroscience, Rutgers University, Piscataway, NJ 08854 USA; 4 Department of Physiology & Biophysics, UC Irvine, Irvine, CA 92697 USA; 5 Sue and Bill Gross Stem Cell Research Center, UC Irvine, Irvine, CA 92697 USA; 6 Department of Physics, Rutgers University, Piscataway, NJ 08854 USA; 7 Molecular Cardiology and Biophysics Division, Victor Chang Cardiac Research Institute, Sydney, NSW Australia; 8 Cancer Pharmacology Research Program, Cancer Institute of New Jersey, Rutgers University, New Brunswick, NJ 08901 USA

**Keywords:** Ion transport, Ion channel signalling, Calcium channels, Membrane structure and assembly

Correction to: *Nature Communications* 10.1038/s41467-022-35034-6, published online 03 December 2022

In this article the affiliation details for Shilong Yang, Boxuan Li, Huan Wang and Zheng Shi were incorrectly given as Department of Chemistry and Chemical Biology, Piscataway, NJ, 08854, USA but should have been Department of Chemistry and Chemical Biology, Rutgers University, Piscataway, NJ 08854, USA.

The affiliation details for Steven Arnold were incorrectly given as Department of Cell Biology and Neuroscience, Piscataway, NJ, 08854, USA but should have been Department of Cell Biology and Neuroscience, Rutgers University, Piscataway, NJ 08854, USA.

The original article has been corrected.

